# High Dynamic Range Imaging at the Quantum Limit with Single Photon Avalanche Diode-Based Image Sensors †

**DOI:** 10.3390/s18041166

**Published:** 2018-04-11

**Authors:** Neale A.W. Dutton, Tarek Al Abbas, Istvan Gyongy, Francescopaolo Mattioli Della Rocca, Robert K. Henderson

**Affiliations:** 1 STMicroelectronics Imaging Division, Tanfield, Edinburgh EH3 5DA, UK; Francesco.Mattiolidellarocca@ed.ac.uk; 2 School of Engineering, The University of Edinburgh, Edinburgh EH9 3JL, UK; tarek.alabbas@ed.ac.uk (T.A.A.); Istvan.Gyongy@ed.ac.uk (I.G.); robert.henderson@ed.ac.uk (R.K.H.)

**Keywords:** single photon avalanche diode (SPAD), high dynamic range, HDR, CMOS image sensor, CIS, single photon counting, SPC, HDR SPC, quanta image sensor, QIS, spatio-temporal oversampling

## Abstract

This paper examines methods to best exploit the High Dynamic Range (HDR) of the single photon avalanche diode (SPAD) in a high fill-factor HDR photon counting pixel that is scalable to megapixel arrays. The proposed method combines multi-exposure HDR with temporal oversampling in-pixel. We present a silicon demonstration IC with 96 × 40 array of 8.25 µm pitch 66% fill-factor SPAD-based pixels achieving >100 dB dynamic range with 3 back-to-back exposures (short, mid, long). Each pixel sums 15 bit-planes or binary field images internally to constitute one frame providing 3.75× data compression, hence the 1k frames per second (FPS) output off-chip represents 45,000 individual field images per second on chip. Two future projections of this work are described: scaling SPAD-based image sensors to HDR 1 MPixel formats and shrinking the pixel pitch to 1–3 µm.

## 1. Introduction

**Figure 1:**
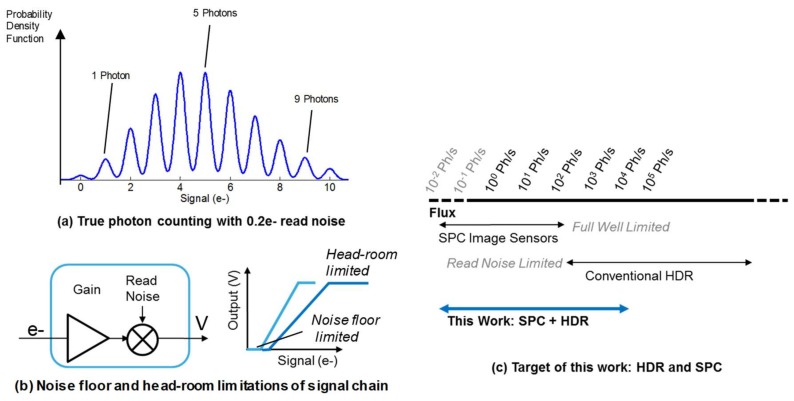
(**a**) Single photon counting is realised in DSERN pixels with <0.30 e^−^ RN. Example is 0.2 e^−^ RN with clearly visible peaks from counted photons during an integration time (**b**) Image sensor analogue signal chains impose both head-room and noise floor limitations on dynamic range (**c**) Target of this work is high dynamic range in a photon counting regime.

Single photon sensitive image sensors offer the ultimate photo-sensitivity to a wide range of applications such as machine vision, scientific imaging, space, defense, and film cameras [1,2,3,4,5]. In all these use cases, high resolution imaging with both high sensitivity and wide dynamic range are required [4]. A range of pixel technologies serve these demands but have their individual limitations. Solid-state Electron Multiplied CCD (EMCCD) and non-solid state solutions such as photo-cathode based Intensified CCD (ICCD), and Intensified CMOS (ICMOS) either offer high sensitivity or Dynamic Range (DR) but not both, given the fundamental noise-floor and head-room limitations of the intensification process combined with the analogue signal chain as illustrated in Figure 1b [6]. Furthermore, none of these technologies directly detects the incident single photons: instead indirectly sensing the electrons from the multiplication or intensification process (which itself brings a range of noise sources).

Truly counting single photons with >90% certainty is realised below 0.3 e^−^ read noise (RN), with an example Deep Sub Electron Read Noise (DSERN) pixel response illustrated in Figure 1a [6]. There are two approaches offering promising solutions for high resolution photon counting image sensors. Firstly, CMOS image sensor (CIS) technology with active pixel sensor readout (APS) has been recently employed in DSERN CIS pixels offering <0.30 e^−^ RN with no electron multiplication process [7,8]. Secondly and the focus of this work, CMOS SPADs offer single photon counting either using analogue techniques or digital logic. However, both CIS and SPAD pixels encounter the same fundamental trade-off between pixel size, dynamic range and sensitivity. Recent research into spatio-temporal oversampling of photon-counting image sensors overcomes the tradeoff of pixel size to full-well or maximum photon count through a range of different techniques [4,6,9].

This paper explores this trade-off using CMOS SPADs and complementary pixel circuits, in an advanced deep sub-micron (DSM) imaging CMOS technology [10], with temporal oversampling to achieve both Single Photon Counting (SPC) and High Dynamic Range (HDR) as shown in Figure 1c with a scalable and compact pixel architecture capable of realising megapixel imaging arrays in the future. We expand on our original works [1,2] with greater detail and further measurements.

## 2. Background

**Figure 2:**
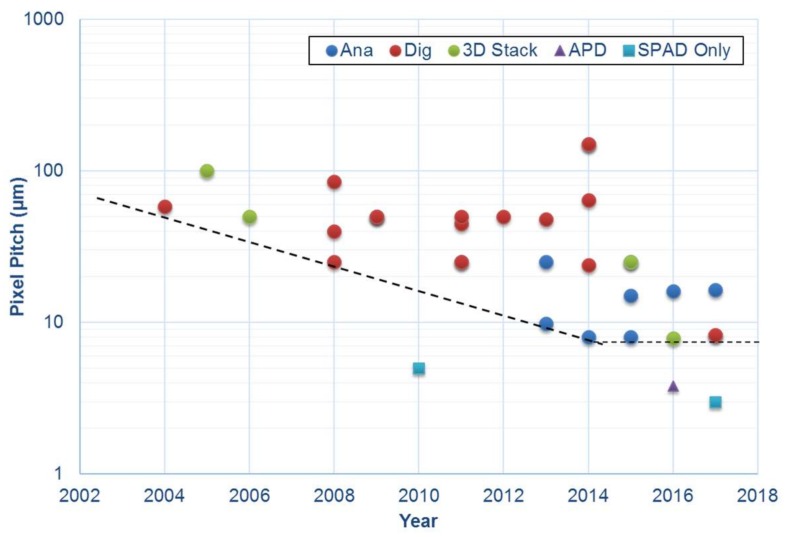
Pixel pitch compared by year of publication for a variety of CMOS SPAD image sensor pixel architectures (analogue or digital monolithic, and all-digital 3D stacked) with a dotted line indicating the trend on reducing pixel pitch now limited at 7.83 µm [12]. Three notable exceptions sit outside this trend, two SPAD diode test structures (SPAD only) at 5 µm [13] and 3 µm pitch [14] and 3.8 µm APD pixel [15].

To achieve high resolution megapixel arrays using avalanche-based pixels, the pixel pitch must be sufficiently small and competitive with the state of the art. For comparison, the leading example of pixel pitch for a CIS SPC pixel is 1.1 micron with 0.22 e^−^ DSERN with a full well in the region of 200 e^−^ in 3D stacked implementation [11]. In contrast avalanche-based pixels are larger for two primary reasons: the pixel circuit is more complex and the APD or SPAD structure itself does not scale readily with technology node. Addressing the latter, scaling down the diode structure is the first pixel design challenge as the device structure requires careful design of the planar high electric field region and guard ring regions providing a transition zone between high and low field regions. Recent examples can be used to illustrate the scaling of APDs and SPADs to achieve compact pixels. Figure 2 shows the recent chronological trend in decreasing pitch of avalanche-based pixels. The pixels are compared for monolithic designs where the majority of smaller pixels are based on analogue circuits due to fewer transistors. This work [2,12] is the first to employ advanced 40 nm CMOS to reduce the pixel pitch of digital photon counting pixels. The black dotted line indicates the trend of pitch reduction. Three data points sit outside of the trend: two lead the field for SPAD pitch (without image sensor pixel circuits) at 5 µm [13] and 3 µm [14], whilst [15] is the first and a remarkable example of a high resolution APD back-illuminated image sensor at 3.8 µm pitch, although with a full-well of only 1 photon. The latter reports two modes of operation: 40 dB dynamic range single photon mode and 60 dB dynamic range CIS mode [15]. Yet neither mode has inherently high dynamic range for SPC. 

The CMOS SPAD is an ideal detector for HDR SPC as it has an intrinsic *DR* greater than 100 dB: capturing photon flux with count rates from ~1 count/second to >10 M count/second for passive recharge (~140 dB dynamic range) and >100 M count/second for active recharge (~160 dB dynamic range). Until recently no SPAD pixel designs, to the knowledge of the authors, had fully utilised the intrinsic HDR of the photo-detector; a first implementation of time-gated pixel with HDR photon counting is very recently published [16].

**Figure 3:**
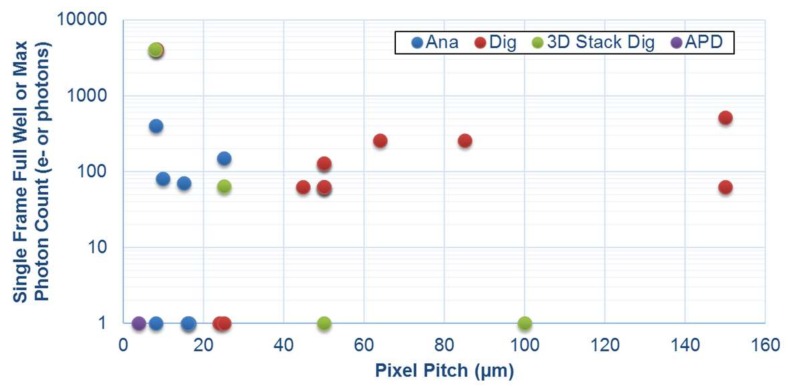
Maximum photon count of SPAD pixels grouped by architecture in comparison to the pixel area.

The pulsing output of the SPAD (or current avalanche of an APD) poses the second pixel design challenge: how to count these pulses in a compact pixel pitch whilst attaining this maximum dynamic range. The three main SPAD image sensor pixel architectures may be considered in relation to the problem: digital counter, analogue counter and 1-bit memory. The simplest architecture is the all-digital ripple counter which is well explored in the literature [17,18]. The counter bit depth is proportional to pixel area and scales readily with CMOS technology node. Alternatively analogue counters (either based on switched current sources [19,20] or charge transfer amplifiers [21,22]) can realise approximately 100 counts in a compact form with reasonable PRNU. The main limitation to increasing the maximum count is the addition of the noise of the analogue signal chain [6]. Furthermore, like all precision analogue circuits in deep sub-micron (DSM) CMOS it does not scale easily with technology node. The 1-bit memory, based on dynamic [23] or static [24] memory structures are the same size as the analogue counter, but record only a single SPAD count so has the lowest maximum count of the three architectures. Yet, it scales much more readily with DSM CMOS technology scaling. Figure 3 highlights the maximum count in comparison to pixel pitch of the three architectures. The most promising in the context of HDR SPC is the digital ripple counter for scaling and functionality.

To overcome the dynamic range limitations of the maximum count of the pixel, two techniques can be combined: HDR imaging and oversampling. Dynamic range enhancement for CIS is well known for over 20 years [25,26]. The most applicable HDR technique is multiple in-pixel memories (or storage nodes) with independent global shuttering providing the benefit of capturing HDR images simultaneously (without multiple sequential exposures) with reduced motion artefacts, for example, for suppression of LED or indoor lighting flicker [27]. Fossum describes HDR oversampling of photon counting image sensors in his theoretical paper on the Quanta Image Sensor (QIS) [4]. Individual binary images (referred to as ‘bit planes’ or ‘field images’) are captured for multiple exposure times then oversampled temporally or spatially to form a HDR frame image. Employing these two concepts together is the basis of our silicon test chip trialing a pixel design capable of HDR. While the sensor used in this work has a limited resolution (96 × 40), its 3D-stacked counterpart [12] is scalable to megapixel arrays and allows pitch reduction with the progress in decreasing technology nodes.

## 3. Silicon Design

**Figure 4:**
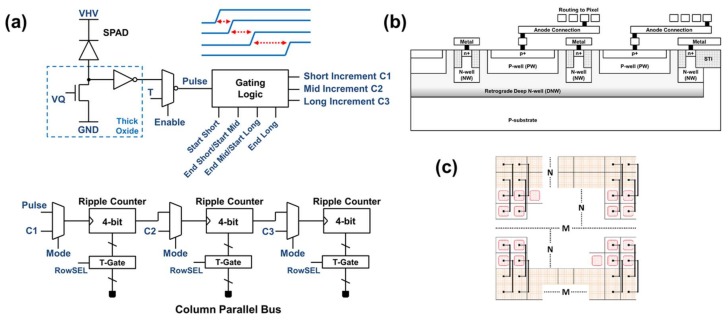
(**a**) Pixel Schematic with dual modes of linear 12 b counter or three 4b counters with individual exposure controls (default mode in this work). (**b**) Cross-section of SPADs shared in a global well (**c**) Top view of SPADs in global well with pixel circuits placed at the edge.

This section describes the design and architecture of our demonstrator IC for HDR SPC in FSI technology. The pixel schematic is shown in Figure 4a. It consists of a SPAD with a single passive quench passive recharge (PQPR) NMOS transistor, four time gating front-end D-type flip flops (in the gating logic block) and 12 toggle flip-flops configurable either as a 12 b ripple counter for linear counting mode or three individual 4 b ripple counters for SPC HDR mode (the default configuration in this work). In linear counting mode, only the first time-gate flip-flop is employed. For HDR mode, the four time-gating D-type flip flops are positive edge triggered where the time-gate integration window is between the rising edges of two gating signals. This provides three contiguous exposure windows ‘short’, ‘mid’ and ‘long’ each with independent in-pixel capture and storage. The time-gating technique is described in [2,12] and provides zero loss of sensitivity around the transition of the three time-gate windows. To provide good matching between the time-gating signals, each is routed through a clock tree at the edge of the array with a driver and line per column.

As described in detail in [2] and shown in Figure 4b in this monolithic implementation the 8.25 µm × 8.25 µm SPAD structure has a p-well (PW) to deep n-well (DNW) junction with retrograde guard ring. The cathode is a shared global DNW permitting 66% fill factor of the anode (considering only the imaging array). The anode is routed to the matching pixel circuit at the edge of the array. The pixel circuits are placed outside of the imaging array and are pitch matched at 8.25 µm × 8.25 µm. In this manner, the pixel circuit is ready for a future 3D-stacked implementation [12].

The integration is global shutter and the rolling all-digital readout is through conventional row-wise timing. Top and bottom readout is employed and the data for each row is sequentially serialised, and each array-half is transmitted off-chip by a single I/O pad at 21.97 Mb/s rate at 1 kFPS. Here we define a frame as the full 12 b data per pixel whether it represents a single 12 b linear mode exposure or an in-pixel summation of 15 binary fields at three different exposures (4 b per exposure) in HDR mode. The data and frame rates are kept intentionally moderate, to understand how this architecture scales as a building block to very high resolution arrays where data-rate and power will be primary limiting factors.

**Figure 5:**
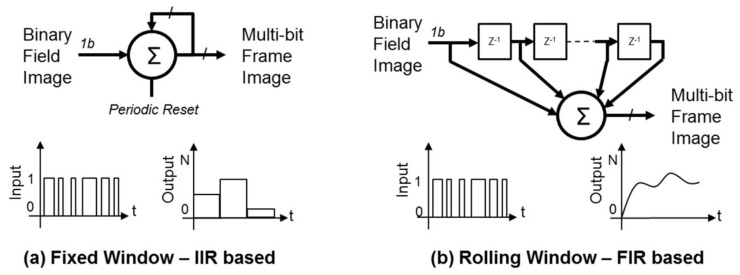
The two primary methods of temporally summing single photon bit planes: (**a**) Fixed averaging based on an IIR filter with periodic reset (i.e., a simple counter). (**b**) Rolling averaging based on FIR filter.

In our previous research [6,9,23,28] into oversampled photon counting, a single bit represents the detection of a photon (the image from the sensor is referred to as a field image or a bit-plane); however, this unary encoding of photon counting is not a power efficient method of data transmission. To address this limitation, some degree of in-pixel summing provides data compression and a power saving. Figure 5 describes the two primary methods of temporally summing bit-planes that can be employed: fixed time window integration (achieved by infinite impulse response (IIR) type filter but periodically reset) [23] and rolling window (finite impulse response (FIR) filter based) averaging [9]. The downside to fixed window summing is the loss of temporal resolution and output frame rate whereas the FIR rolling average provides temporal resolution at the input bit-plane frame rate but comes with higher power, data rate and area costs. Fixed summing in-pixel is easily implemented, and provides data compression. It is clear that a trade-off is made of data compression versus temporal resolution and frame rate. Furthermore, this problem is intensified when implementing HDR with multiple exposures. In this work, a compromise is chosen to sum up to 15 bit planes in pixel for each of the HDR exposures using each 4b counter. In effect this is a 3.75 times data compression and power saving (15 unary bits to 4 binary bits), at the cost of a 15 times reduction in temporal resolution by fixed IIR filter summing.

**Figure 6:**
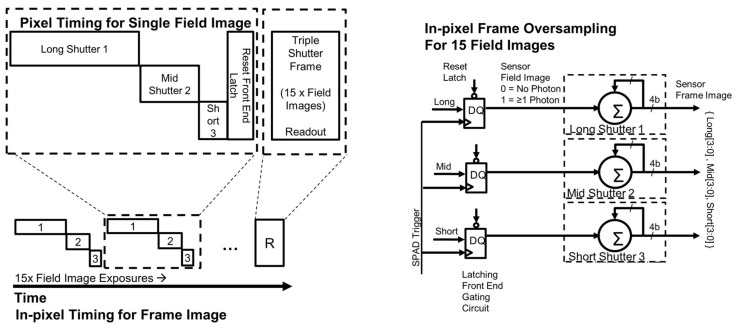
(**Left**) HDR SPC pixel timing diagram with three field images with different shutter times (long, mid, short) each capturing a single bit (representing ≥1 photon), 15 field images are summed in pixel before column parallel readout. (**Right**) pixel functionality block diagram showing the latching front end and the 3 ripple counters temporally summing 15 field images per readout frame.

The pixel timing is illustrated on the left of Figure 6. To create the HDR image, three exposures (short, mid and long) are captured. Ideally for conventional HDR timing [27], the exposures are interleaved to minimise motion blur, but due to the front end circuit these are captured back to back. However, this effect is considered to be minimal in our implementation of SPC HDR as the three exposures are captured within micro-seconds of each other. QISs capture 1 b per field image (representing ≥1 photon) and, here in the HDR QIS, 1 b is captured for each exposure. After the three exposures are completed, the front end latching circuit is reset for the next field image and the in-pixel ripple counters are incremented as shown on the right of Figure 6. Once 15 field images have been summed in-pixel (compressed to 4 b) to constitute one frame, the 12 b data represents 45 field (or bit plane) exposures. The data is readout via all-digital column parallel readout at 1000 frames per second (FPS) i.e., the sensor operates at 45,000 fields per second (FiPS).

## 4. Measurements Results

**Figure 7:**
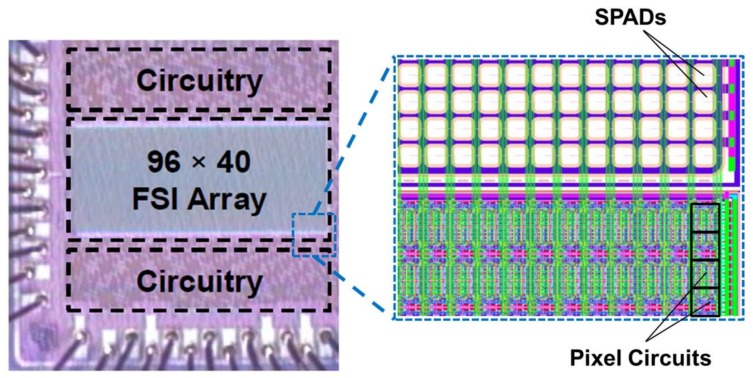
(**Left**) Photomicrograph of the fabricated IC. (**Right**) Top layout view showing the SPADs shared in a global well and pitch-matched pixel circuits in a bank below.

The 96 × 40 imager was fabricated in STMicroelectronics 40 nm FSI imaging technology. A photomicrograph and layout view is shown in Figure 7. The test chip measures 1.0 mm × 1.0 mm. The SPADs are in a single global shared well and the pixel circuits, at the same pitch, are at the periphery. This test array allows the oversampled HDR capability to be evaluated.

**Figure 8:**
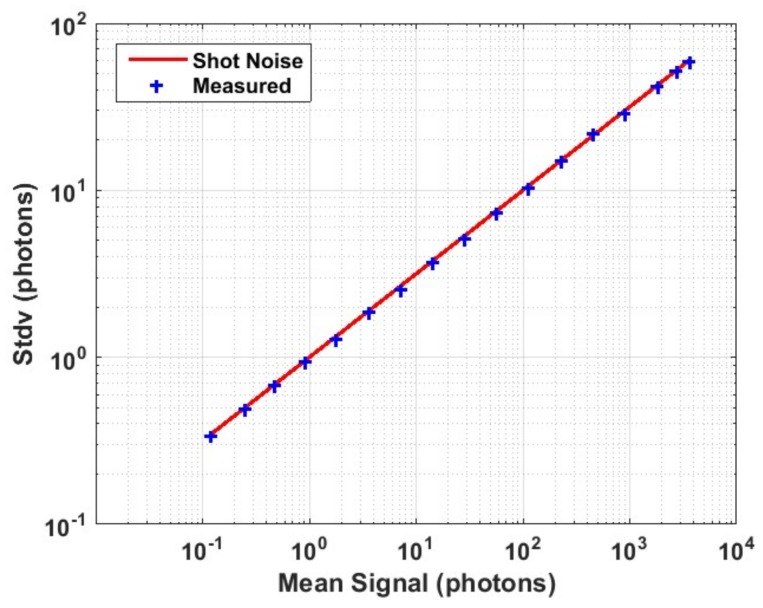
Photon transfer curve for single pixel in linear counting mode indicating purely shot noise limited single photon counting in this image sensor. The red line is a model of the shot-noise limit.

Figure 8 illustrates the photon transfer curve (PTC) of a single pixel in linear counting mode to confirm that the photon counting mechanism of the SPADs and the image sensor is entirely shot-noise limited. The red-line is a model of shot-noise limited SPC and there is minimal deviation of measured results from the ideal model.

To demonstrate the sensor’s quanta response the current through an LED source has been swept while data has been captured at a variety of exposure settings. For each light point, a total of 50 bit planes or fields of 96 × 40 pixels were spatially and temporally combined to result in a total of 192,000 ensembles ‘M’. For the purpose of speeding up the measurement all of the 96 × 40 pixels where spatially summed to contribute towards the total number of ensembles, while in a practical QIS use case a smaller subset of pixels or jots (8 × 8 for example [11]) would be spatially summed to represent one image element. The bit density ‘D’ vs. the input signal ‘H’ curves were produced by dividing the total number of counts at each light point by M.

**Figure 9:**
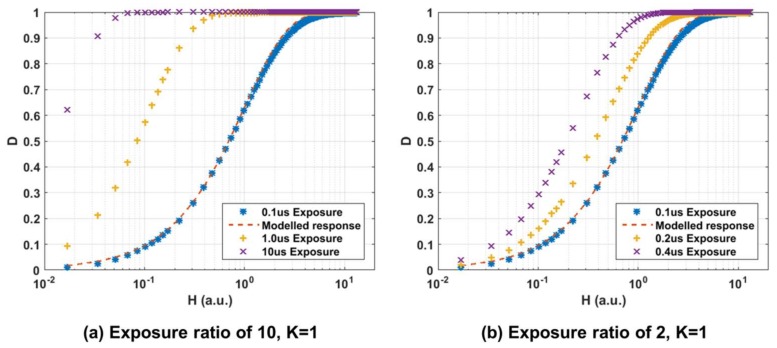
Measured normalized intensity (D or ‘Bit plane density’) to normalized input signal (H) for two sets of integration times for 1 photon threshold (K = 1). (**a**) Exposure ratio of 10 with Short = 100ns, Mid = 1 μs, Long = 10 μs. (**b**) Exposure ratio of 2 with Short = 100 ns, Mid = 200 ns, Long = 400 ns.

Figure 9 shows the measured QIS response for a photon threshold ‘K’ of 1 where a pixel is assigned a binary value of ‘0’ for no photons detected and a binary value of ‘1’ for one or more photons detected.

This binary assignment is performed by the in-pixel gating and counting logic depicted in Figure 4. Two scenarios have been explored where three different exposures of ratios of 10 (0.1, 1 and 10 µs) and ratios of 2 (0.1, 0.2 and 0.4 µs) were used. The *x*-axis has been normalised such that an input signal of *H* = 1 yields a bit density *D* = 0.63 for the shortest exposure setting of 0.1 µs. This is known as the ‘full exposure’ point as defined by [4]. The 0.1 µs exposure setting has been chosen as the reference as it is the common setting across all measurements to follow. The modelled QIS response for this exposure is shown as the dashed red line where *D* is defined as:

(1) $$D=1-e^{-H}$$

The measured data exhibits some deviation from the ideal model which could be attributed to non-linearity in the light source output power, illumination non-uniformity, photo-response non-uniformity and temporal variations as measurements were acquired over hours which would all contribute to the error in the spatio-temporally oversampled data. Nevertheless, the measured data offers a qualitative insight into QIS behavior. As can be seen from the results of the longest exposure setting of 10 µs, it was not possible to reach low bit density values due to the limitations in the illumination source used. The authors opted for not combining data acquired by using different neutral density filters to avoid adding in more error.

**Figure 10:**
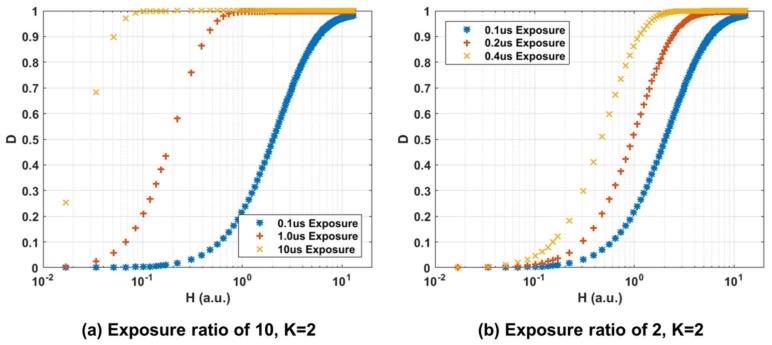
Measured normalized intensity (D or ‘Bit plane density’) to normalized input signal (H) for two sets of integration times for 2 photon threshold (K = 2). (**a**) Exposure ratio of 10 with Short = 100 ns, Mid = 1 μs, Long = 10 μs. (**b**) Exposure ratio of 2 with Short = 100 ns, Mid = 200 ns, Long = 400 ns.

The measurement was repeated for an emulated photon threshold of K = 2 (pixel assigned a binary value of ‘0’ for no photons or one photon detected and binary value of ‘1’ for two or more photons detected) by using linear counting mode (12 bit) and three sequential exposures. This emulation is necessary due to the latching single bit (K = 1) front end in HDR mode. Fifty single frames (no on-chip summation) were captured for each exposure setting where each pixel exhibits photon counts between 0 and 4095. By post processing the captured intensity frames the pixel values were re-assigned to transform the frame into a binary bit-plane or field. In the future an improved pixel design with multi-photon triggering could achieve the variable K threshold in-pixel. This variable threshold adjusts the non-linear intensity to exposure characteristic which is an interesting property of the QIS. The same exposure ratio settings were used and DlogH curves are shown in Figure 10.

To evaluate the dynamic range (*DR*) and signal-to-noise ratio (SNR) of the quanta image sensor, and following from the theory presented in [4], *DR* hereby defined as:

(2) $$DR=20\times \log\left(\frac{H_{m}}{H_{n}}\right)$$

where *H_m_* is the *H* value at which the measured signal reaches 99% of its saturation limit and *H_n_* is the *H* value equivalent to the noise level (read + dark). Since the used digital sensor has no read noise as shown in Figure 8, the only contribution to *H_n_* is from the dark count rate (DCR) of the SPADs. For all carried measurements the SPADs were biased at 2 V excess voltage for which the median DCR is ~150 cps at room temperature [2]. Using Equation (1), and taking D to be 150 cps × 0.1 µs, the equivalent *H_n_* is calculated to be 1.5 × 10^−5^. This value was used for all *DR* calculations in this work while *H_m_* was estimated from the wanted measured signal.

It is worth noting that the number of ensembles ‘M’ has an effect on *DR* as the minimum observable signal is one photon per M ensembles (or 1/M), so for the maximum *DR* (*DR_max_*) to be achieved it is necessary that the used number of ensembles is greater than the noise floor equivalent (i.e., M > 1/D(*H_n_*)), else the *DR* will be limited by the ability to observe a signal. Since M of 192,000 used in the presented measurements satisfies this condition, all *DR* figures reported herein represent *DR_max_* which might not be achievable in a practical QIS scenario.

For SNR calculations an alternative ‘exposure referred SNR’ or *SNR_H_* definition was proposed by [4]. The objective of this definition is to project the SNR as measured in the *y*-axis (bit density D or ‘voltage referred’) onto the input *x*-axis (H). The reason behind this is that the voltage referred SNR will result in an artificial increase due to the compression of data by the QIS response and so *SNR_H_* is a more meaningful measure. *SNR_H_* is defined as:

(3) $$SNR_{H}=\frac{H}{\sigma_{H}}$$

where *σ_H_* is defined as:

(4) $$\sigma_{H}=\sigma_{Tot}\frac{dH}{dM_{Tot}}$$

**Figure 11:**
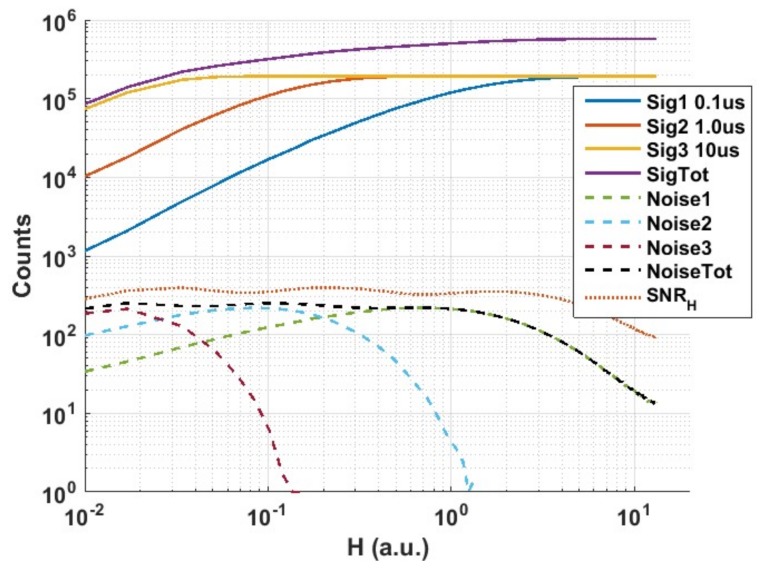
Measured signal, noise and *SNR_H_* responses for 3 exposure settings with exposure ratio of 10 and photon threshold K = 1.

Figure 11 shows the cumulative QIS signal response and *SNR_H_* for photon threshold K = 1 and three different exposures with a ratio of 10 (0.1 µs, 1 µs and 10 µs). *Sig*1, *Sig*2 and *Sig*3 are the counts *M*_1_, *M*_2_ and *M*_3_ of the three corresponding exposures. *SigTot* (or *M_Tot_*) is the linear summation of the counts of the three responses:

(5) $$SigTot=Sig1+Sig2+Sig3=M_{Tot}=M_{1}+M_{2}+M_{3}$$

*Noise*1 is the standard deviation of *Sig*1 and under the assumption of Poisson statistics is given by:

(6) $$Noise1=\;\sigma_{1}=\sqrt{\frac{\left(M-M_{1}\right)\times M_{1}}{M}}$$

where *M* is 192,000 (50 fields × 96 × 40 pixels). *Noise*2 and *Noise*3 are defined similarly and *NoiseTot* is the total noise of the cumulative response and is defined as:

(7) $$NoiseTot=\sigma_{Tot}=\sqrt{{\left(\sigma_{1}\right)}^{2}+{\left(\sigma_{2}\right)}^{2}+{\left(\sigma_{3}\right)}^{2}}$$

Hence it is possible to calculate *SNR_H_* for the measured data from the above equations. While it is not possible to observe the rise of *SNR_H_* at low *H* values due to the measurement setup limitations and the fact that the long 10 µs exposure response masks the response from the shorter exposures at these low *H* values, it is interesting to see how *SNR_H_* peaks forming a ‘plateau’ region with very smooth transitions or ‘ripples’ when data from different exposures are summed as opposed to the dips in SNR observed in conventional image sensors. Using the equations above, *SNR_H_* and *DR* have been calculated for cases of single, double and triple exposures with a ratio of 10 showing how *DR* increases from ~70 dB to more than a 100 dB in this example (Table 1).

**Figure 12:**
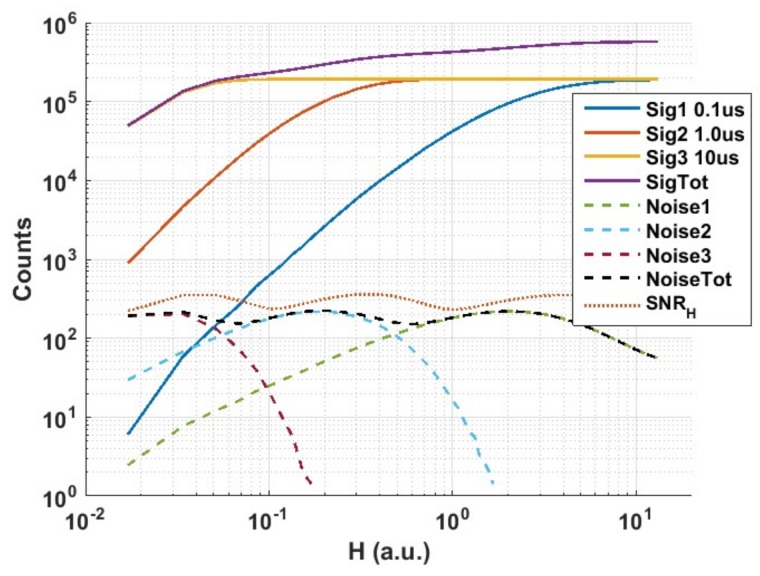
Measured signal, noise and *SNR_H_* responses for 3 exposure settings with exposure ratio of 10 and photon threshold K = 2.

The same analysis was repeated for the measurements of the same exposure settings with a photon threshold of K = 2 to see the effect of photon threshold on *SNR_H_* and *DR* in the case of multi-photon single-bit pixels. The signal and noise plots are shown in Figure 12 and *SNR_H_* and *DR* are summarised in Table 2. It is observed that while the *DR* increases slightly above that of K = 1 this comes at the expense of more pronounced ripples or variation in *SNR_H_* at the plateau region when combining the three exposures. The measured *SNR_H_* variation in this example was ~2 dB. The increase in *DR* is attributed to the fact that the QIS response for K = 2 (Figure 10a) is shifted to the right with respect to the response for K = 1 (Figure 9a) moving the 99% saturation point further while the lower end of the response is still dominated by the noise floor. Moreover, the K = 2 response exhibits a steeper slope compared to that of K = 1 which reflects on the transition between the three exposure settings and hence higher variation in *SNR_H_*.

Another factor that has been investigated is the effect of the exposure ratio on *SNR_H_* and *DR*. For that, the same measurements as above were repeated for K = 1 and exposure ratios of 2 (0.1 µs, 0.2 µs and 0.4 µs), 4 (0.1 µs, 0.4 µs and 1.6 µs), 6 (0.1 µs, 0.6 µs and 3.6 µs) and 8 (0.1 µs, 0.8 µs and 6.4 µs). The 0.1µs exposure setting is the common factor across all experiments. The measured *SNR_H_* and *DR* for all cases are summarised in Table 3. It is observed that while *SNR_H_* slightly decreases as the exposure ratio increases, *DR* is unaffected. This suggests that the *DR* extension is dominated by the shortest exposure setting which in this example was the common 0.1 µs. Of course this holds true due to the fact that the minimum observable signal is dominated by the noise floor as a very large number of ensembles has been used as explained previously. For a smaller number of ensembles the minimum detectable signal will then be determined by the longest exposure setting and hence influence the achievable *DR*. In a rolling shutter sensor the shortest exposure would be dominated by line time and in a global shutter sensor it is down to signal drivers and acceptable temporal aperture ratio. The *SNR_H_* peak is higher for smaller exposure ratios because as can be seen from Equations (3) and (4), *SNR_H_* is dependent on the rate of change in the total signal which is higher for short exposure ratios as the individual responses are close to each other and add up together more rapidly (i.e., *dM_Tot_/dH* is higher for shorter exposure ratios). On the other hand, for longer exposure ratios the individual responses are spaced apart resulting in a slower rate of change in the total signal as they are summed together.

**Figure 13:**
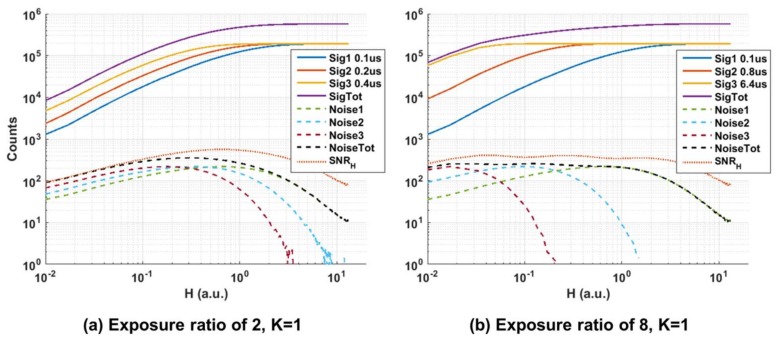
Measured signal, noise and *SNR_H_* response for 3 exposure settings and K = 1. (**a**) Exposure ratio of 2. (**b**) Exposure ratio of 8.

While it is possible to obtain a high *DR* response with a single short exposure the advantage of having longer exposures is apparent when comparing the *SNR_H_* response for exposure ratios of 2 and 8 (Figure 13). Both cases result in a *DR* of ~108 dB but as the exposure ratio increases (2 to 8) the *SNR_H_* response results in a wider plateau region spanning a larger portion of the input signal H.

The presented results show how the dynamic range of a single frame triple-exposure sensor can be increased which is also an improvement over our previous paper [1] which required two frames to capture the three sub-exposures for the dynamic range extension. While other QIS sensors can attain similar *DR* performance, the partial in-pixel field summation providing 3.75× data compression and the ability to capture multiple exposure settings simultaneously significantly reduces readout requirements and offers better immunity against motion artifacts as compared to other works.

**Figure 14:**
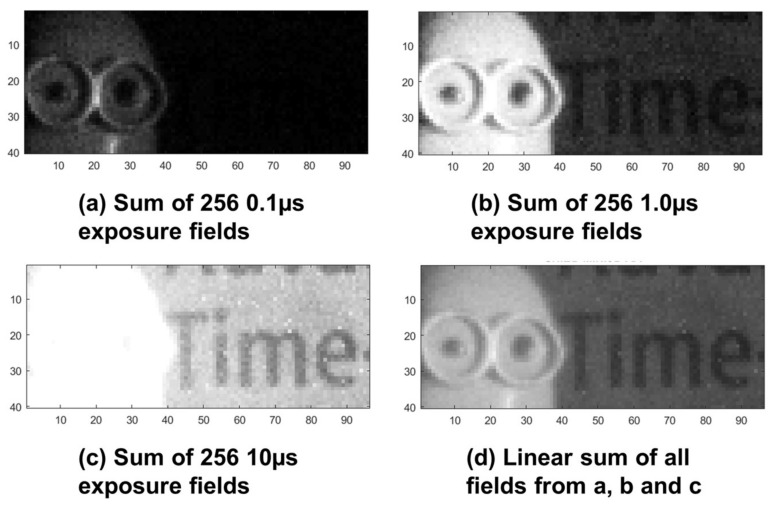
Images captured by 96 × 40 FSI sensor [2]. (**a**) Sum of 256 fields at 0.1 µs exposure, a Minion figure is visible; (**b**) Sum of 256 fields at 1.0 µs exposure, the Minion is visible but slightly overexposed while faint letters appear in the background; (**c**) Sum of 256 fields at 10 µs exposure, the Minion is totally overexposed but the letters appear clearer; (**d**) Linear sum of all the 768 fields from a, b and c to form an HDR image preserving all details.

**Figure 15:**
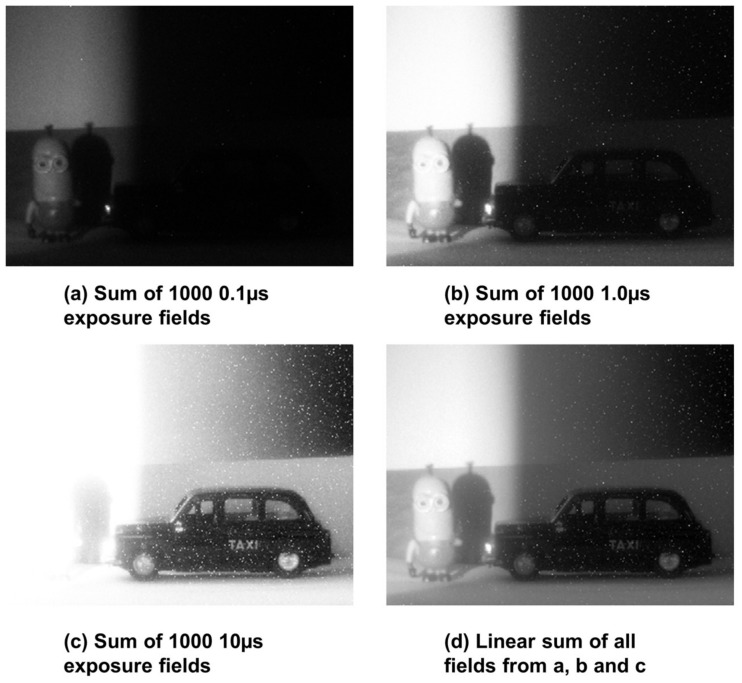
Images captured by 320 × 240 SPC sensor from [23]. (**a**) Sum of 1000 fields at 0.1 µs exposure, a Minion appears in the lit portion of the scene; (**b**) Sum of 1000 fields at 1.0 µs exposure, the Minion is slightly overexposed but a car figure appears in the dark region of the scene; (**c**) Sum of 1000 fields at 10 µs exposure, Minion is completely overexposed but more detail of the car is apparent. Notice that high DCR pixels appear as white dots. (**d**) Linear sum of all the 3000 fields from a, b and c to form an HDR image preserving all details.

The 96 × 40 sensor is used to capture a high dynamic range scene as a demonstration of HDR QIS in operation in Figure 14. To demonstrate this proof of principle further, Figure 15 shows images captured by the 320 × 240 SPC imager from [23] which has higher resolution, wider field of view and lower DCR. Both sensors were operated with a photon threshold of K = 1 and different exposures were acquired sequentially as only static scenes were imaged.

The example given in Figure 14 allows for a brief benchmarking of HDR QIS performance. The presented analysis in this work shows that for the given 96 × 40 sensor it is possible to achieve a maximum dynamic range (*DR_max_*) of 108 dB. 

Yet the *DR* of the example in Figure 14 is limited by the number of ensembles (M = 256) rather than the noise floor, so the effective *DR* (*DR*_effective_) is limited by the minimum observable signal (bit density D = 1/256 for each exposure). To calculate *DR*_effective_ the equivalent *H* value for this minimum signal can be calculated from Equation (1), and using that as the denominator in Equation (2) results in an effective *DR* of 99.6 dB for a three exposure (0.1 µs, 1 µs and 10 µs) scenario showing the effect of M on achievable *DR*.

## 5. Discussion and Future Projections

The multi-megapixel QIS is an attainable goal for both CIS and avalanche-based detectors. Each has their own advantages: the CIS-based and APD-based have low pixel pitches but rely upon conventional active pixel sensor (APS) analogue readout so can only oversample block-by-block [11] not per-pixel. SPAD pixels have the challenge of shrinking the pixel pitch but have the benefit of all-digital pixels allowing a wide range of in-pixel functionality such as HDR oversampling. Table 4 evaluates recently published APD and SPAD-based image sensor architectures projected up to a 1 M pixel HDR oversampled SPC image sensor with at least three HDR field exposures. For this study, the output frame rate is set at 240 FPS with 256 field images summed per frame. The single bit architectures require significant data rate and power to achieve this specification. Furthermore, as they can only capture one field before readout, readout time is presumed to be a significant limiting factor in their sensitivity. In contrast, this work permits up to 15 bit planes summed in-pixel (so needing 17 readouts to obtain the oversampling ratio (OSR) of 256) which is a tradeoff between temporal resolution (for imaging fast moving objects or fast phenomena) and data rate. The analogue counter in [29] allows up to 80 bit planes to be summed but would suffer from very high motion artifacts if implementing sequential HDR timing and requiring 12 readouts (four per exposure). The downside to all the sensors based on SPAD, is the pixel pitch remains high so the array dimensions are large.

This is addressed in the second future projection for SPAD-based pixel circuits. Table 5 presents the maximum count (or equivalent full well) of this work for a single linear exposure and for HDR dual and triple exposures. 3D-stacked SPAD image sensors have been demonstrated [12,30], and this study indicates the future path to decrease the pitch of these image sensors’ bottom tier pixel circuit can be based on all digital ripple counter and leverage the shrink gained through use of future technology nodes. Assuming the challenge of shrinking the SPAD diode and the stacking interconnect can also be met, then below 20 nm CMOS should realise SPAD pixels with HDR capability and pitch in the 1–3 µm range. In all these cases, spatio-temporal oversampling is required as the in-pixel bit depth is low. While this work relies only on temporal (summing fields) oversampling, spatio-temporal (summing fields and groupings of pixels) oversampling was adopted by other works to create an image from a quanta image sensor for example by summing an 8 × 8 × 8 kernel (pixels × pixels × fields) [11].

## 6. Conclusions

This paper examines methods to best exploit the HDR of the SPAD in a high fill-factor photon counting image sensor that is scalable to megapixel arrays. The digital ripple counter split into 3 individual exposures allows HDR photon counting to be realized in a compact pixel pitch. In-pixel summing provides data compression to increase the on-chip frame rate while maintaining low data rates off-chip.

The future expansion of array sizes of SPAD image sensors relies upon both compact pixel architectures and the shrink of pixel circuit areas. With 3D stacking of top-tier SPADs on advanced digital DSM CMOS, pixel pitches below 8 µm are possible. The recent advent of three tier stacking [31] allowing the interconnection of back-illuminated sensing layer, processing and memory layers will further enhance the dynamic range, frame rate and power of both SPAD-based and CIS-based oversampled image sensors while facilitating novel ISP approaches such as motion tracking and solid-state optical image stabilization [28].

## Figures and Tables

**Table 1:** Calculated *SNR_H_* and *DR_max_* from measured data for the cases of single, double and triple exposures with a ratio of 10 and K = 1.

Exposure	*SNR_H_* (dB)	*DR_max_* (dB)
10 µs	50.5	72
10 µs + 1 µs	51.8	90
10 µs +1 µs + 0.1 µs	52	109

**Table 2:** Calculated *SNR_H_* and *DR_max_* from measured data for the cases of single, double and triple exposures with a ratio of 10 and K = 2.

Exposure	*SNR_H_* (dB)	*DR_max_* (dB)
10 µs	50.7	75
10 µs + 1 µs	50.9	92.7
10 µs +1 µs + 0.1 µs	51.1	115.8

**Table 3:** Measured *SNR_H_* and *DR_max_* for a three exposures scenario and K = 1 with different exposure ratios of 2 (0.1 µs, 0.2 µs and 0.4 µs), 4 (0.1 µs, 0.4 µs and 1.6 µs), 6 (0.1 µs, 0.6 µs and 3.6 µs) and 8 (0.1 µs, 0.8 µs and 6.4 µs).

Ratio	*SNR_H_* (dB)	*DR_max_* (dB)
**2**	55	108
**4**	53.5	108
**6**	52.7	108
**8**	52.2	108
**10**	52	109

**Table 4:** Projection of different APD and SPAD pixel architectures to 1MPixel HDR QIS with ≥ 3 HDR field exposures.

	This Work	[23]	[1,12]	[29]	[24]	[15]
Pixel Pitch	8.25	8	7.83	15	24	3.8
Circuit Type	Digital Ripple Counter	NMOS Dynamic Memory	Digital Ripple Counter	Analogue Counter	NMOS Static Memory	APD + CIS APS
Oversampling in-pixel	✓	✕	✓	✓	✕	✕
Exposures In-Pixel	3	1	2	1	1	1
Counter Depth	4b	1b	6b	7b	1b	1b
Summing in-pixel per Exposure	15	1	63	80	1	1
Pixel data output width	12b	1b	12b	7b	1b	1b
Projection to 1MPix (1024 × 1024) 3D Stacked QIS with >100 dB HDR						
Array Dimension (µm)	8448	8192	8017	15,360	24,576	3891
QIS Output Frame Rate (FPS)	240	240	240	240	240	240
Total OSR per Frame	256	256	256	256	256	256
Sensor Field Rate (FiPS) *	4096	184,320	2926 **	2304	184,320	184,320
Interface Data Rate (Gpbs)	48	180	34.3	15.8	180	180
In-pixel Data Compression Ratio ***	3.75	None	10.5	11.4	None	None
Motion Artifact	Best–V.Low	High	Low	V.High	High	High
Multiple HDR exposures in-pixel	✓	✕	✓	✕	✕	✕

* Field Rate = [OSR] × [Required Frame Rate] × [3 HDR Exposures]/([Exposures in Pixel] × [Bit Planes in Pixel]). ** N.B. For 2 exposures in pixel, 2 readouts are required for 3 HDR exposures. *** Compression Ratio = [Bit Planes in Pixel] × [Exposures In Pixel]/[Output Data Width].

**Table 5:** Projected pixel shrink trends, of 2D monolithic and 3D-stacked SPAD photon counting pixels based on an all-digital ripple counter architecture, towards 3D-stacked SPAD or Avalanche based Quanta Image Sensors with multi-megapixel arrays.

CMOS Tech‘ Node (nm) for Digital Logic	Circuit Pixel Pitch (µm)	2D Monolithic	3D Stacked	Single Exposure Linear Counter		Dual Exposure HDR		Triple Exposure HDR	
				Max Count	Counter Bit Depth	Max Count	Counter Bit Depth	Max Count	Counter Bit Depth
This Work, [1,12]									
40	7.83–8.25	✓	✓	4095 ^1^	12	63	6	15	4
Future Projected Trends									
32	6	✓	✓	16,383 ^1^	14	127	7	15	4
22	3		✓	127	7	7	3	3	2
16	1.5		✓	7	3	1	1	1	1
11	1.0		✓	7	3	1	1	1	1
11	0.75		✓	1	1	-	-	-	-

^1^ With the exception of these two high maximum counts, spatio-temporal oversampling is required as the in-pixel max count (or equivalent full well) is low.
